# ITGA5 Promotes Tumor Progression through the Activation of the FAK/AKT Signaling Pathway in Human Gastric Cancer

**DOI:** 10.1155/2022/8611306

**Published:** 2022-09-24

**Authors:** Jun-fu Wang, Ye-yang Chen, Si-wen Zhang, Kun Zhao, Yue Qiu, Ye Wang, Jian-cheng Wang, Zhu Yu, Bo-pei Li, Zheng Wang, Jun-qiang Chen

**Affiliations:** ^1^Department of General Surgery, The First Affiliated Hospital of Nanchang University, Nanchang 330031, China; ^2^Department of Gastrointestinal Surgery, The First Affiliated Hospital of Guangxi Medical University, 6 Shuangyong Road, Nanning, 530021 Guangxi Zhuang Autonomous Region, China; ^3^Department of Gastrointestinal Surgery, The First People's Hospital of Yulin, Yulin, 537000 Guangxi Zhuang Autonomous Region, China; ^4^Guangxi Key Laboratory of Enhanced Recovery after Surgery for Gastrointestinal Cancer, Nanning, Guangxi Zhuang Autonomous Region, China

## Abstract

**Background:**

ITGA5 is an adhesion molecule that integrates the intracellular structures with the extracellular matrix to perform biological functions. However, ITGA5 is highly expressed in a variety of tumors and is involved in tumor progression by promoting cell proliferation and metastasis. Nevertheless, little research has been performed on its function in gastric cancer. Therefore, the aim of this study was to investigate the role of ITGA5 in gastric cancer, focusing on the mechanism regulating the proliferation, invasion and migration.

**Methods:**

The expression of ITGA5 in gastric cancer tissues was assessed by the use of molecular bioinformatics databases and high-throughput sequencing of gastric cancer tissues from patients. Western blot, qPCR, and immunohistochemistry were performed to detect the expression of ITGA5 in samples from gastric cancer patients and gastric cancer cell lines. Furthermore, the ITGA5 gene was silenced and overexpressed in gastric cancer cells, and the effect on proliferation, invasion, migration, and tumorigenic ability was assessed.

**Results:**

ITGA5 mRNA and protein expression were upregulated in gastric cancer cell lines and tissues from patients, and its expression was closely associated with tumor size, lymph node metastasis, and TNM stage. *In vitro* and *in vivo* experiments showed that ITGA5 silencing resulted in the inhibition of proliferation, invasion, migration, and graft growth of gastric cancer cells; conversely, the overexpression resulted in the promotion of these cell functions. Our results finally showed that the effect of ITGA5 on proliferation, invasion, and migration of gastric cancer cells was performed through the activation of the FAK/AKT pathway.

**Conclusions:**

ITGA5 promotes proliferation, invasion, and migration of gastric cancer cells through the activation of FAK/AKT signaling pathway, suggesting that ITGA5 may be potentially considered as a new target in gastric cancer therapy.

## 1. Introduction

Gastric cancer is one of the most common gastrointestinal malignancies with global incidence, with more than one million new cases each year, especially in East Asian countries, where the incidence and mortality rates are among the highest. Global cancer incidence and mortality statistics released in 2020 by the International Agency for Research on Cancer ranked gastric cancer fifth in the global cancer incidence spectrum. Gastric cancer accounts for 5.6% of the total incidence and the fourth in the mortality spectrum. It also accounts for 7.7% and 58.3% of cancer deaths in Asia, which represents 59.5% of the global cancer population [1]. The global cancer burden continues to grow and poses a great public health challenge, with a greater increase in our country compared to Europe and the USA. The statistics from the 2020 China Cancer Report show that stomach cancer ranks third in incidence and mortality rate [1]. What is worse is that most patients are already in the middle and late stages when they are diagnosed with gastric cancer, and the five-year survival rate is less than 30%, with poor surgical and chemotherapeutic treatment [2]. Early screening, diagnosis, and treatment are key factors to improve the prognosis of gastric cancer patients; thus, new means of cancer prevention should be developed, and different targets should be considered to ensure an effective intervention. In addition, multiomics research should be relied upon to explore cancer-related markers, so as to effectively reduce cancer burden and provide new directions for the diagnosis and treatment of gastric cancer.

Integrins are heterodimeric transmembrane glycoproteins formed by two subunits, *α* and *β*. They are mainly expressed on the surface of cell membranes and mediate cell-cell, cell-extracellular matrix, and cell-pathogen interactions [3]. The effect of integrins on tumors is mainly through the cytokines, ligands, and angiogenesis [4]. ITGA5 is one of the many members of the integrin family; it is a receptor for fibronectin that influences extracellular matrix and intracellular signaling and is closely associated with the development of many tumors [5, 6]. Several studies identified ITGA5 as a prooncogene, playing an important role in the proliferation, apoptosis, invasion, and metastasis of a variety of malignancies [7–9]. However, the available information regarding ITGA5 in gastric cancer is poor, and the relationship between ITGA5 and gastric cancer is still unclear. Our group found that ITGA5 was highly expressed in gastric cancer samples from patients, as revealed by bioinformatics analysis in the early stage, and it was closely related to the activation of the extracellular matrix (ECM), and focal adhesion kinase (FAK), as well as AKT and PI3K signaling, suggesting that ITGA5 might be considered as a potential biomarker and therapeutic target for gastric cancer. ITGA5 in gastric cancer is only predicted by bioinformatics data, without basic experimental confirmation; thus, this study further explored it role by an in-depth study using *in vivo* and *in vitro* experiments.

FAK adherent plaque kinase is a nonreceptor tyrosine kinase associated with the integrin signaling pathway that is enriched in adherent plaques and, together with Src kinase, coordinates adhesion turnover, actin cytoskeleton dynamics, and cell morphology to regulate tumor cell migration and invasion [10, 11]. FAK plays an important role in integrin and tyrosine kinase-mediated signaling pathway [12]. PI3K/AKT signaling pathway is involved in the development of many common cancers [13]. FAK can be activated by ITGA5, which in turn triggers the activation of PI3K/AKT signaling pathway [14]. Thus, the in-depth study of FAK might help to deeply understand the mechanism of gastric carcinogenesis, providing a new target against the invasive metastasis of gastric cancer.

This study revealed that ITGA5 was significantly upregulated in gastric cancer tissues of patients, and its expression was negatively correlated with patient survival. The effect of ITGA5 on gastric cancer cells was examined by *in vivo* and ex vivo experiments, so as to understand its role. The results showed that ITGA5 is an oncogene in gastric cancer, allowing the progression of gastric cancer by the activation of the FAK/AKT signaling pathway. Therefore, ITGA5 may be a potential new target in gastric cancer therapy.

## 2. Materials and Methods

### 2.1. High-Throughput Sequencing of Patient Tissue Samples

High-throughput sequencing was performed on 10 gastric cancer tissues and corresponding paraneoplastic normal tissue samples from patients hospitalized at the Shanghai Biotechnology Co. The differential analysis of genes expressed in gastric cancer tissues and corresponding paracancerous normal tissues resulting in a *q* value <0.05 and difference multiplicity Log|FoldChang| > 2, considered as screening conditions, allowed the discovery of the significantly changed genes, and bioinformatics analysis was performed.

### 2.2. Bioinformatics Database Data Acquisition

The expression of the ITGA5 gene was detected by GEPIA software (http://gepia.cancer-pku.cn/) and ENCORI Pan-Cancer Analysis Platform software, after the selection of the normal samples and tumor samples from the TCGA database, using the Log2FC > 0.5 and *P* value <0.05 as the screening condition to explore the expression of ITGA5 in gastric cancer tissues. All the expression data were converted to Log2 (TPM+1) for analytical mapping. The Kaplan-Meier plotter was used to perform the prognostic analysis of ITGA5: the K-M plotter database (http://kmplot.com/analysis) data were obtained from GEO, EGA, and TCGA. The overall survival (OS), postprogression survival (PPS), and relapse-free survival (RFS) of patients with increased expression of ITGA5 was mapped using the mRNA-seq data from TCGA and gene chip data from GEO (GSE14210, GSE15459, GSE22377, GSE29272, and GSE51105和GSE62254). The GSEA desktop software application (http://www.broadinstitute.org/gsea/index.jsp) was used to further explore the potential value of the ITGA5 gene in biological processes and pathways to investigate its potential mechanisms. Enrichment analysis by GSEA was considered statistically significant when *P* < 0.05 and FDR < 0.25.

### 2.3. Patients and Sample Collection

All tissue samples were collected from 130 gastric cancer patients admitted to the Department of Gastrointestinal Glandular Surgery of the First Affiliated Hospital of Guangxi Medical University and the Department of Gastrointestinal Surgery of Guangxi Provincial Cancer Hospital from September 2006 to December 2019, with preoperative pathological confirmation based on electronic gastroscopy. All patients had normal liver and kidney function, while the patients who had preoperative radiotherapy and those with other systemic tumors or tumor metastasis to the stomach were excluded from this study. Postoperative pathology was confirmed by oncologic pathologists. Tumor tissues and the corresponding paracancerous normal tissues (>5 cm from the cancerous tissues) were collected; the tissue samples were quickly frozen at -80°C and stored for subsequent experiments. Additional samples were fixed in 10% formaldehyde, subsequently embedded in wax blocks, and cut into 4-*μ*m-thick slides. Immunohistochemical staining for ITGA5 was performed as described in the paragraph 2.11. The study was approved by the ethical review committees of the two hospitals, and written informed consent was signed by the patients. The specimens were histologically classified according to the classification system of the Union for International Cancer Control (UICC) 8th edition.

### 2.4. Cell Culture

Human gastric cancer cell lines (AGS, MKN28, and HGC27) and normal gastric mucosal epithelial cells Ges-1 were purchased from the Shanghai Cell Bank, Chinese Academy of Sciences and Shanghai Fuxiang Biotechnology Co. The culture medium was supplemented with 10% fetal bovine serum, 1% penicillin, and 1% streptomycin, and the cells were incubated at 37°C under 5% CO_2_.

### 2.5. Real-Time Quantitative Polymerase Chain Reaction (qRT-PCR)

ITGA5 mRNA expression in the adjacent tissues, gastric cancer tissues, and gastric cancer cell lines was detected by qRT-PCR. Total RNA was extracted using the Nucleozol Total RNA Extraction Kit (MACHEREY-NAGEL, Germany), and its concentration and purity were detected by Nanodrop ND-2000. The expression levels of different mRNAs were detected by Takara Biotechnology Ltd. RNA was reverse transcribed using a reverse transcription kit. qRT-PCR was performed using SYBR Green I fluorescence detection kit (Takara, China), and the quantitative analysis was performed using 7500 real-time PCR system. The primers were synthesized by Shanghai Bioengineering Technology Co., Ltd., and the sequences were as follows: target gene ITGA5, forward: 5′-GGCTTCAACTTAGACGCGGAG-35′- CACCCTGTTGCTGTAGCCAAA-3′; reverse: 5′-TGGCTGGTATTAGCCTTGGGT-3′; internal reference GAPDH, forward: 5′- TGACTTCAACAGCGACACCCA-3^″^; reverse: 5′- CACCCTGTTGCTGTAGCCAAA-3′; The reaction conditions were as follows: 95°C pre-denaturation for 30 s, 95°C for 10 s, 60°C for 30 s, 35 cycles. The experiment was performed using 3 replicates.

### 2.6. Western Blotting

Changes in the expression of the following proteins, such as ITGA5, FAK, and AKT, were detected by Western blotting. Proteins were lysed using RIPA lysis buffer (Thermo Fisher Scientific) and protease/phosphatase inhibitor (Beyotime Biotechnology), and their concentrations were detected by BCA (Thermo Fisher Scientific). Proteins were separated by 10% polyacrylamide gel electrophoresis, transferred to a PVDF membrane (GE Healthcare, Piscataway, NJ), and blocked with 5% skimmed milk powder for 1 h at room temperature. The membrane was then incubated overnight with primary antibodies on a 4°C shaker. The antibodies and concentrations used were the following: ITGA5 (Proteintech, 1 : 1000 dilution), FAK (Cell Signaling Technology, 1 : 1000 dilution), p-FAK (Cell Signaling Technology, 1 : 2000 dilution), AKT (Cell Signaling Technology, 1 : 1000 dilution), p-AKT (Cell Signaling Technology, 1 : 1000 dilution), and GAPDH (Cell Signaling Technology, 1 : 10000 dilution). Next, the membrane was incubated with the secondary antibody (Cell Signaling Technology 1 : 5000 dilution) at room temperature for 1 hr. Finally, the membrane was treated with ECL developer (Pierce), and the bands were developed using a gel imager FluorChem (USA, ProteinSimple).

### 2.7. Constructs and Vectors

ITGA5 silencing and overexpression plasmids were designed and synthesized by GeneCopoeia (Guangzhou, China). Three silencing plasmids (shRNA-1, shRNA-2, and shRNA-3) were constructed, and the sequence with the highest silencing efficiency was used for the subsequent experiments. The plasmids were transfected into gastric cancer cells using Lipofectamine 3000 (Invitrogen, USA) with sh-NC and OE-NC as negative controls, respectively.

### 2.8. Cell Proliferation Assay

Cells in good growth state in their log phase were used. A total of 4 × 103 cells were counted and seeded into 96-well plates. 10 *μ*L CCK8 solution was added into each well at 0, 24, 48, 72, and 96 h after seeding; the cells were incubated at 37°C and 5% CO2 for 2 h; and then, the absorbance was measured at 450 nm with an enzyme marker. As regard the clone formation assay, 400 cells were collected and seeded into culture dishes, and the culture was terminated when the clones were visible to the naked eye in the dishes. The cells were fixed with 4% paraformaldehyde, stained with crystal violet; the clones were observed under the microscope; and those formed by each group of cells were calculated using ImageJ software.

### 2.9. Cell Invasion and Migration Assay

BD Falcon Cell Culture Inserts (#3422) were purchased from BD (Franklin Lakes, NJ, USA) and were used for the migration assay. BD BioCoat™Matrigel™ Invasion Chamber (BD, #3422) was used for the invasion assay according to the manufacturer's instructions. The cells were incubated at 37°C in a humidified atmosphere containing 5% CO2 for 24 h, and then invaded or migrated to the bottom surface of the membrane, which were fixed with 95% alcohol. Next, the cells were fixed with 4% paraformaldehyde and stained with crystal violet. The number of cells passing through the chambers was observed and counted under the microscope and the total number of cells traversed was counted. Five fields of view were counted and their mean values were calculated.

### 2.10. Animal Model

Healthy male BALB/c nude mice (4 weeks, *n* = 8) were purchased from Nanjing Junke Bioengineering Co., Ltd. and maintained in specific pathogen free environment. The nude mice were randomly divided into OE-NC group and OE-ITGA5 group (*n* = 4), while OE-ITGA5 stable AGS cells and OE-NC cells as a control group were subcutaneously injected into the right foreleg area. The tumor volume was measured, and the body weight was evaluated every 5 days for 5 weeks. After 35 days, the nude mice were anesthetized by an intraperitoneal injection and placed under a live imager (PerkinElmer, IVIS Lumina III, Shanghai) to take pictures. Some samples of the tumor tissues were fixed in 4% paraformaldehyde for immunohistochemistry, and some others were stored at -80°C for further use. All experimental procedures were approved by the Animal Ethics Committee of Guangxi Medical University.

### 2.11. Immunohistochemistry

Gastric cancer tissue sections from patients and nude mouse tumor tissue sections were treated with xylene and alcohol, treated with EDTA antigen repair solution, blocked with 3% H_2_O_2_ for 30 min, and incubated with goat serum for 30 min. Next, the slides were incubated with ITGA5 antibody (Proteintech, 1 : 200 dilution) overnight at 4°C. The slides were then incubated with the secondary antibodies at room temperature for 30 min, nuclei were stained with DAPI, and the slides were imaged and photographed under a fully automated fluorescent microscope. Results were scored independently by two pathologists and blinded for ITGA5 staining.

### 2.12. Statistical Analysis

Statistical analysis was performed using SPSS 22 and Graphpad Prism 7.0 statistical software, and the results were expressed as mean± standard deviation (±*s*). Student *t*-test was used for the comparison between two groups, and one-way analysis of variance (one-way ANOVA) was used for comparison among multiple groups. The cardinality test was used for data counting. All experiments were repeated at least three times, and a value of *P* < 0.05 was considered statistically significant.

## 3. Results

### 3.1. ITGA5 Is Highly Expressed in Gastric Cancer Tissues and Cell Lines

**Figure 1:**
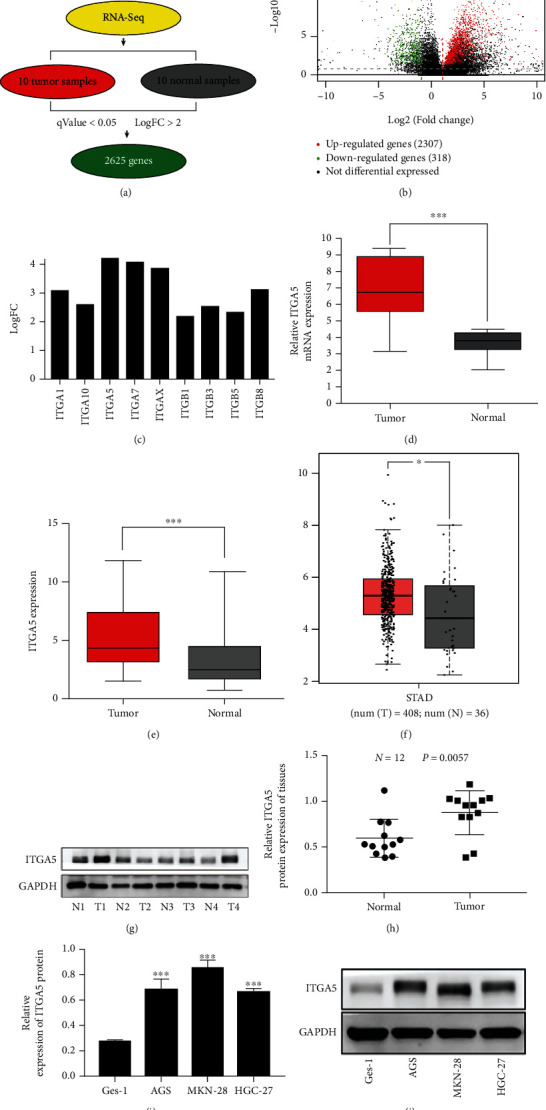
Relative expression of ITGA5 in gastric cancer tissues and cell lines. (a). mRNA RNA sequencing of gastric cancer tissues and corresponding paracancerous normal groups identified a total of 2625 differentially expressed genes. (b). Volcano plot showing 2307 upregulated genes and 318 downregulated genes among the differentially expressed genes, with the red color representing the upregulated genes and the green color representing the downregulated genes. (c). Log FC>2 as the criterion to screen out significantly differentially expressed integrin family member profiles, among which ITGA5 expression differential fold was the largest. (d) RNA sequence screened out ITGA5 expression in gastric cancer tissues versus the corresponding paracancerous normal tissues (*N* = 10). mRNA expression level of ITGA5 was significantly upregulated in gastric cancer tissues (*P* < 0.001). (e) ITGA5 expression in 40 gastric cancer tissues and corresponding paraneoplastic tissues by qRT-PCR. ITGA5 mRNA expression was significantly upregulated in gastric cancer tissues (*P* < 0.001). (f) ITGA5 mRNA expression in 408 gastric cancer tissues and 36 corresponding paraneoplastic tissues by TCGA database. ITGA5 mRNA is highly expressed in tumor tissues. (g–h) ITGA5 protein expression in gastric cancer tissues and corresponding paracancerous normal tissues determined by Western blot. ITGA5 expression in most gastric cancer tissues was higher than that in paracancerous tissues using GAPDH as a loading control (*N* = 12, *P* = 0.005). (i–j) ITGA5 expression in three gastric cancer tissues and three gastric cancer cell lines determined by qRT-PCR (i) and Western blot (j). ITGA5 mRNA and protein expression were upregulated in gastric cancer cells compared with its expression in the normal gastric mucosal epithelial cell line Ges-1. The high expression of ITGA5 in gastric cancer was found in high-throughput sequencing and bioinformatics databases, and this result was verified in clinical samples and gastric cancer cell lines.

mRNA high-throughput RNA sequence was performed on 10 matched pairs of gastric cancer tissues and paracancerous normal tissues from patients, and the differentially expressed genes were identified by *q* value <0.05 and difference fold |FoldChang| > 2. A total of 2625 differentially expressed mRNAs were identified (Figure 1(a)). Among them, 2, 307 are upregulated and 318 are downregulated, as shown in the volcano plot in Figure 1(b), in which red represents the upregulated genes and green represents the downregulated genes. ITGA5 expression was the highest among the significantly differentially expressed integrin genes and was significantly highly expressed in gastric cancer tissues. Thus, it was selected as the target gene for our study (Figure 1(c)). A total of 10 pairs of gastric cancer sequencing samples showed a significantly higher ITGA5 mRNA expression compared to the expression in the corresponding paracancerous normal tissues (Figure 1(d)). The RNA-seq data in the TCGA database is analyzed by platform software to confirm this result, revealing that the ITGA5 mRNA was upregulated in the 408 GC tissues compared to the 36 normal gastric epithelial tissues (*P* < 0.05), as shown in Figure 1(f). Subsequently, the expression of ITGA5 in gastric cancer tissues was confirmed by qRT-qPCR and Western blot, and the results showed that ITGA5 was significantly higher in gastric cancer tissues than in the adjacent normal tissues (Figures 1(g) and 1(h)). Next, the expression of ITGA5 mRNA and protein in gastric cancer cell lines was examined, resulting in a higher expression in all the three gastric cancer cell lines than in the Ges-1 cells, with a higher expression in AGS and MKN28 cells (Figure 1(i) and 1(j)). Thus, these two cell lines were selected for subsequent experiments. The above results indicated that ITGA5 was highly expressed in gastric cancer tissues and cells.

### 3.2. Increased ITGA5 Expression Is Associated to Poor Prognosis in Gastric Cancer Patients

**Figure 2:**
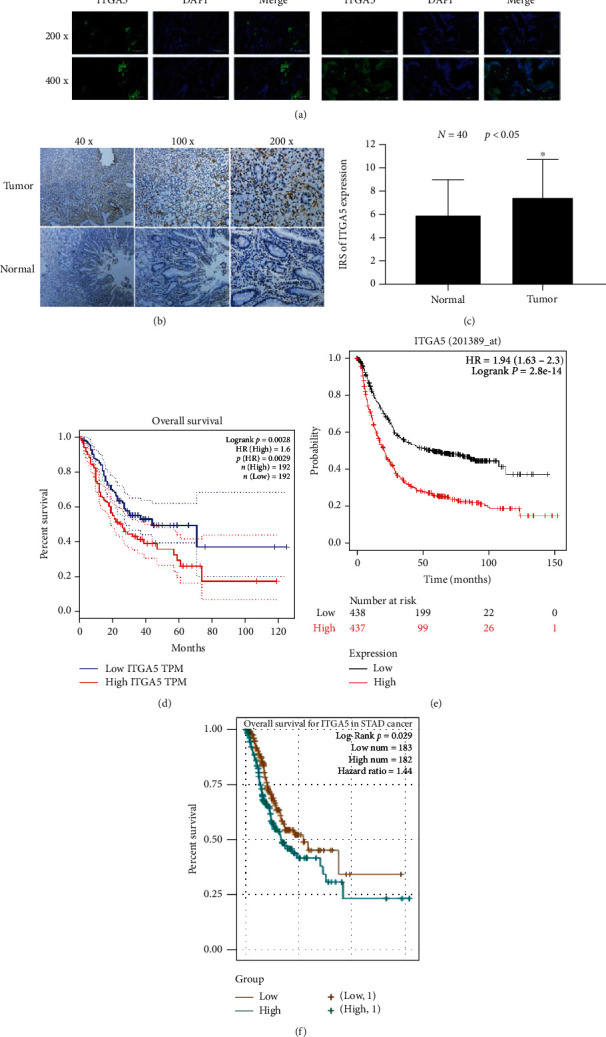
ITGA5 expression in the tissues of gastric cancer patients correlated with prognosis. (a). Immunofluorescence images of ITGA5 expression in tumor tissues and their paracancerous tissues showing its high expression in the tumor, mostly in the cell membrane. (b). Immunohistochemical images of ITGA5 protein expression in 40 gastric cancer tissues and 40 paracancerous normal tissues showing its high expression in the tumor, mostly in the cell membrane. (c–e) The results were analyzed by GEPIA platform software, Kaplan-Meier plotter public data platform, and starbase platform Sun Yat-sen University network public database. All the three databases showed that ITGA5 gene expression was correlated with prognostic factors of gastric cancer patients; patients with high ITGA5 expression had a poor prognosis; and patients with low ITGA5 expression had a better prognosis. ITGA5 is highly expressed in gastric cancer. Its high expression level is negatively correlated with the prognosis of gastric cancer patients.

ITGA5 protein expression was significantly increased in gastric cancer tissues from patients, as revealed by tissue immunofluorescence, and ITGA5 was mainly expressed on the cell membrane (Figure 2(a)). The comparison of ITGA5 expression in 40 pairs of gastric cancer tissues with the paired paraneoplastic tissues detected by immunohistochemistry revealed that ITGA5 expression was significantly increased in gastric cancer tissues compared with the paired paraneoplastic tissues (*P* < 0.05) (Figure 2(c)). Further comparison of the clinicopathological data of 130 gastric cancer patients showed that ITGA5 expression was not significantly correlated with age, gender, and histological staging. However, ITGA5 expression was significantly correlated with tumor size, lymph node metastasis, and TNM pathological stage (*P* < 0.05). ITGA5 expression was increased in gastric cancer patients with larger tumors compared with smaller tumors (*P* < 0.05). ITGA5 expression was increased in gastric cancer patients with lymph node metastasis compared with no lymph node metastasis (*P* < 0.05). ITGA5 expression was significantly increased in advanced gastric cancer compared with early gastric cancer (*P* < 0.05) (Table 1). The software databases GEPIA, Kaplan-Meier, and ENCORI Pan-Cancer Analysis Platform were analyzed to explore whether ITGA5 could predict the survival of gastric cancer patients, and all three databases showed that the prognostic survival of gastric cancer patients was shorter when ITGA5 expression was increased (Figures 2(d)–2(f)), suggesting that ITGA5 gene plays an important role in gastric cancer.

### 3.3. ITGA5 Promotes the Proliferation, Invasion, and Migration Ability of Gastric Cancer Cells In Vitro

**Figure 3:**
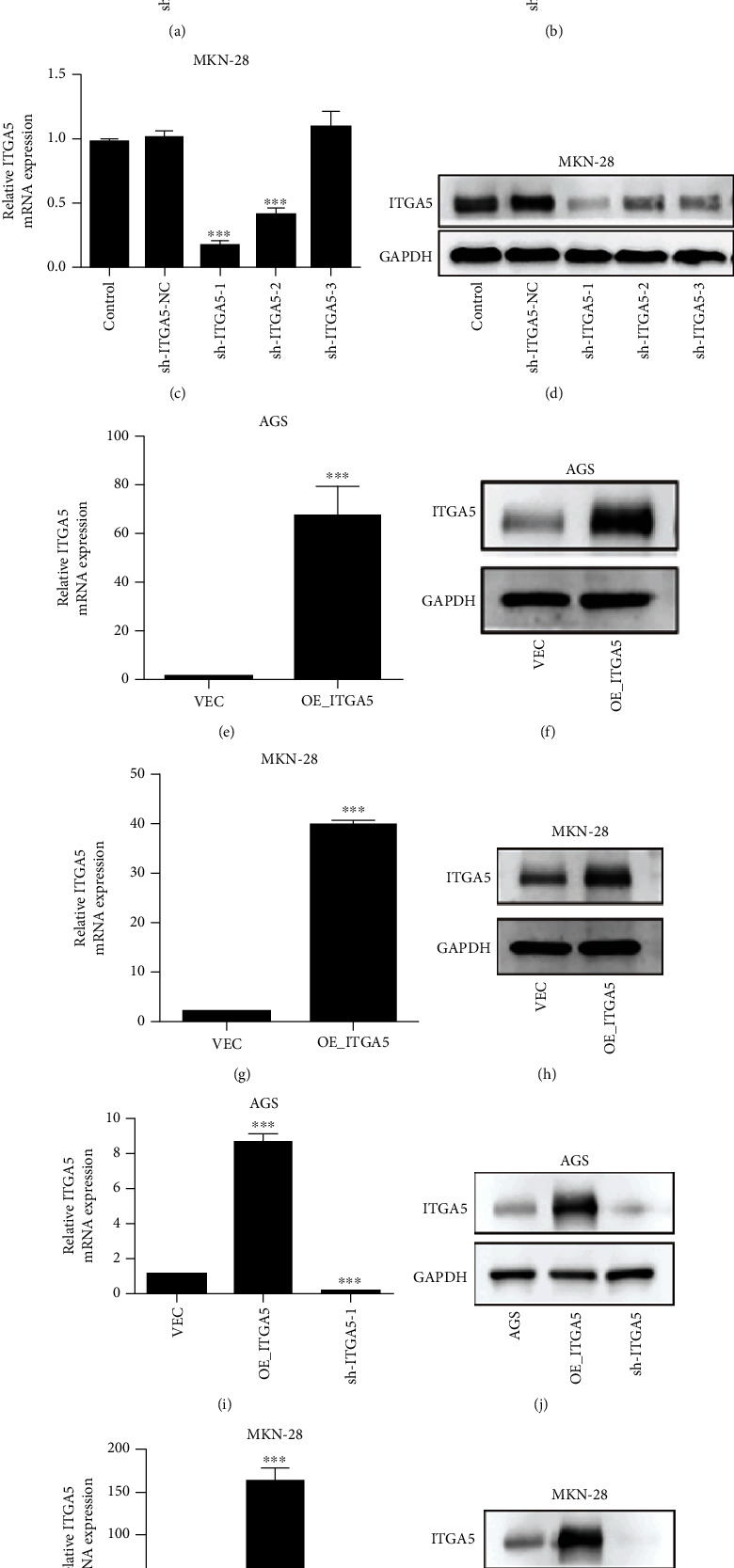
ITGA5 plasmid screening and lentiviral cell line construction for stable transfection. (a–d) Expression of the silenced ITGA5 was significantly reduced in AGS and MKN-28 cells compared to control by qRT-PCR and Western blot. (e–h) Successful overexpression of ITGA5, since it was significantly increased in AGS and MKN-28 cells. (i–l) Stable transfection of sh-ITGA5 expression in two gastric cancer cell lines after transfection with ITGA5 and OE-ITGA5 plasmids (^∗∗^*P* < 0.01; ^∗∗∗^*P* < 0.001 by *t*-test). Screening the plasmid of ITGA5 gene and constructing a stable cell line.

**Figure 4:**
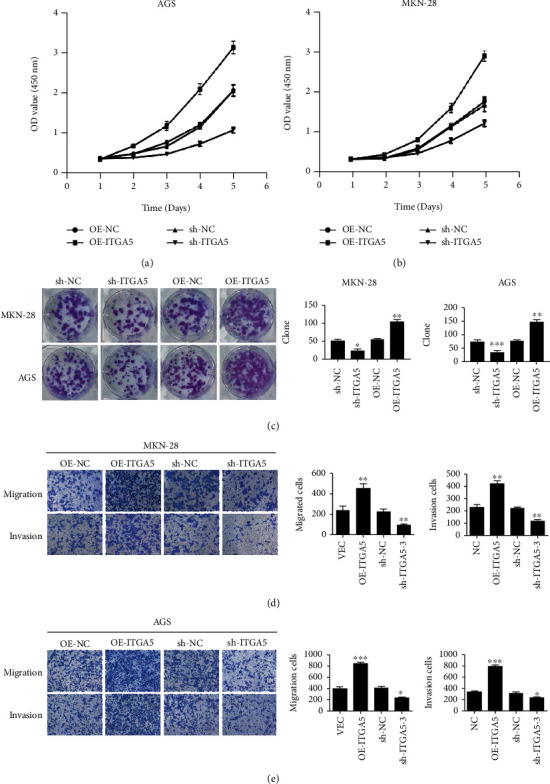
Effect of ITGA5 on proliferation, invasion and migration of gastric cancer cells. (a–b) Effect of ITGA5 on the proliferation of gastric cancer cells by CCK8 cell proliferation assay. ITGA5 overexpression promoted the proliferation of AGS and MKN-28 cells, while ITGA5 silencing inhibited the proliferation of AGS and MKN-28 cells. (c) ITGA5 expression promoted the clonogenic ability of AGS and MKN-28 cells (100× magnification). ITGA5 silencing inhibited the clonogenic ability of AGS and MKN-28 cells (100× magnification). (d–e) ITGA5 overexpression promoted the invasion and migration ability of AGS and MKN-28 cells (100× magnification). ITGA5 silencing inhibited the invasion migration ability of AGS and MKN-28 cells (100× magnification). Results are expressed as mean ± SD of 3 independent experiments (^∗^*P* < 0.05; ^∗∗^*P* < 0.01; ^∗∗∗^*P* < 0.001 by *t*-test). ITGA5 gene promotes the growth of subcutaneous tumor in nude mice in vivo.

AGS and MKN-28, 2 gastric cancer cell lines with relatively high ITGA5 expression, were selected for this experiment to further understand the biological function of ITGA5 in gastric cancer. shITGA5-1 and OE-ITGA5 effectively silenced and overexpressed ITGA5, respectively, in both cell lines. qRT-PCR and Western blotting confirm the results, as shown in Figures 3(a)–3(h). Lentivirus-mediated stable cell lines of shITGA5-1 and OE-ITGA5 were further constructed. The results of qRT-PCR and Western blotting suggest that the construction of the silencing and overexpression of ITGA5 stable cells was successful, as shown in Figures 3(a)–3(c). CCK-8 and clone formation assays showed that ITGA5 silencing significantly inhibited the proliferation and clone formation of gastric cancer cells. In contrast, ITGA5 overexpression significantly increased the proliferation and clone formation of gastric cancer cells. Transwell invasion and migration assays showed that ITGA5 gene silencing significantly decreased the invasion and migration ability of gastric cancer cells. In contrast, ITGA5 gene overexpression significantly promoted the invasion and migration ability of gastric cancer cells (Figures 4(a)–4(i)). These results suggested that the ITGA5 gene promotes the proliferation, invasion, and migration of gastric cancer cells *in vitro*.

### 3.4. ITGA5 Promotes the Tumorigenic Ability of Gastric Cancer Cells

**Figure 5:**
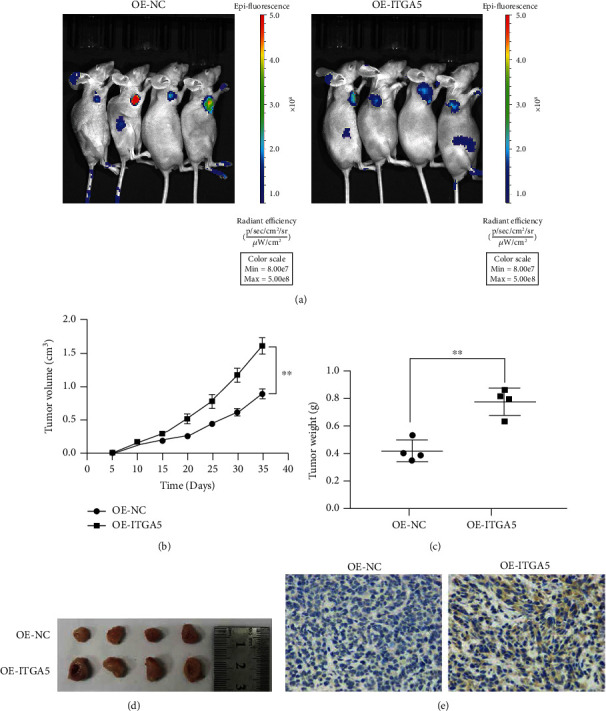
ITGA5 promoted the growth of gastric cancer cells *in vivo*. (a) Subcutaneous tumor live imaging in nude mice. (b–e) Subcutaneous injection of AGS cells NC and AGS overexpressing ITGA5 into nude mice (*N* = 4); the tumor volume was measured every 5 days (b). Excision of the tumors 35 days after inoculation of the cells and their weight (c). Tumors photographed after their removal (d). Immunohistochemical staining revealing ITGA5 expression in transplanted tumors (^∗^*P* < 0.05; ^∗∗^*P* < 0.001; ^∗∗∗^*P* < 0.001). ITGA5 gene can affect the biological behavior of gastric cancer cells and promote the proliferation, invasion and migration of gastric cancer cells.

AGS stable-transformed cells were inoculated into nude mice to construct a nude mouse tumor model to further verify in vivo the role of ITGA5 in gastric cancer. The subcutaneous masses were palpable after 7 days of tumor cell injection, and the weight of the mice and volume of the tumor in the two groups of animals were recorded every five days. The nude mice were treated after 35 days, and biopsy was performed. The effect of overexpression of ITGA5 on tumor progression was examined, and the live images showed the successful construction of the nude mouse transplantation tumor model for gastric cancer (Figure 5(a)). The experimental results showed that the OE-ITGA5 group had a significantly faster tumor growth, larger tumor volume and significantly increased graft tumor weight than the NC group (*P* < 0.05). These results suggested that the tumorigenic ability of tumor cells also increased when the ITGA5 expression in gastric cancer cells inoculated in nude mice was increased. ITGA5 protein expression in the tumor tissues of animals in the NC group and ITGA5 overexpression group was detected by immunohistochemistry, and the results showed that ITGA5 was significantly increased in the tumor tissues of the ITGA5 overexpression group (Figures 5(e) and 5(i)). These results confirm the lentiviral transfection efficiency and the tumor growth effect exerted by ITGA5. This result was also consistent with our in vitro experiments and clinical results.

### 3.5. ITGA5 Activates FAK/AKT Signaling Pathway

**Figure 6:**
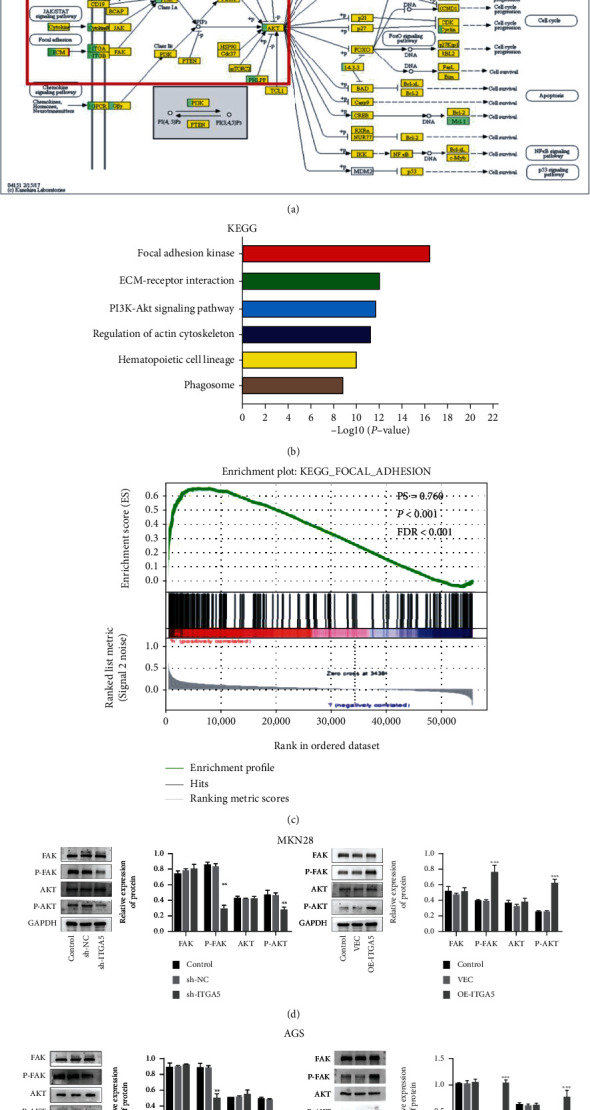
ITGA5 regulated the biological process of gastric cancer cells by activating the FAK/AKT signaling pathway. (a–b) Tissue sequencing results showing that ITGA5 was involved in the signaling pathways involving focal adhesion kinase (FAK), extracellular matrix (ECM), and PI3K-AKT-related pathways. (c) GSEA enrichment analysis revealing that ITGA5 abnormal expression was closely associated with FAK. PS = 0.76, *P* < 0.001, FDR < 0.001. (d–e) p-FAK detected by western blot in AGS and MKN-28 cells silenced with ITGA5; p-AKT expression was significantly reduced, while FAK and AKT expression did not change significantly. (f–g) p-FAK detected in AGS and MKN-28 cells overexpressing ITGA5; p-AKT expression was significantly increased, while FAK and AKT did not change significantly (^∗∗^*P* < 0.01; ^∗∗∗^*P* < 0.001 by *t*-test). High-throughput sequencing and bioinformatics data were used to predict the signal pathway and biological process involved by ITGA5, and Western Blot was used to further verify the possible mechanism of ITGA5 affecting the biological behavior of gastric cancer cells.

The FAK/AKT signaling pathway plays a crucial role in the proliferation, invasion, migration, and adhesion of gastric cancer. The signaling pathways involved in ITGA5 were analyzed based on high-throughput sequencing data and GSEA enrichment analysis. The results showed that the mainly enriched signaling pathways involving ITGA5 were focal adhesion, ECM-receptor interaction, PI3K-AKT, and cancer-related pathways (Figures 6(a)–6(c)). Therefore, the role of FAK/AKT/PI3K signaling pathway in the oncogenic effect of ITGA5 was further explored. Thus, ITGA5 silencing and overexpression on FAK/p-FAK, AKT/p-AKT, and PI3K/p-PI3K protein expression in two gastric cancer cell lines were evaluated by Western Blot. The results showed that the silencing of the ITGA5 gene significantly downregulated p-FAK and p-AKT expression (*P* < 0.05), but not FAK, AKT, PI3K and p-PI3K compared with the control and negative control groups. In contrast, the overexpression of ITGA5 gene resulted in a significant upregulation of p-FAK and p-AKT expression (*P* < 0.05), but FAK, AKT, PI3K and p-PI3K were not significantly modified compared with the control and negative control groups (Figures 6(d) and 6(e)). The above results indicated that the ITGA5 gene activated the FAK/AKT signaling pathway, suggesting that ITGA5 could affect the progression of gastric cancer by regulating the expression of p-FAK and p-AKT proteins.

## 4. Discussion

Integrins are heterodimers present on the surface of nucleated cells, and under normal conditions, they are mainly distributed on the cell membrane. However, a small amount of integrins belonging to a family of adhesion molecules is also localized in the cytoplasm and is able to integrate intracellular structures with the extracellular matrix to perform biological functions [15, 16]. Dimeric complexes of different integrins can bind to the same ligand, and the biological behavior of the cell is determined by the expression pattern of cell surface integrins [17]. Integrins form dimers in the endoplasmic reticulum, and they are translated and modified in the Golgi apparatus, and subsequently translocate to the cell surface, where they bind to the extracellular matrix upon activation. This binding causes the bind of skeletal proteins to integrins, phenomenon that is affecting cell proliferation, invasion, migration, polarization, and differentiation. Thus, integrin-induced dysregulation of cell adhesion and signaling is a prerequisite for many tumorigenesis [18–23].

ITGA5 is a member of the integrin family, with a molecular weight of approximately 130 KD. In recent years, more and more tumors with modified expression of ITGA5 have been reported, attracting interest in carrying out in-depth research. Several studies showed that ITGA5 is expressed in several organs of the human body, but its expression varies among different organs and is highly expressed in a variety of human tumors, such as esophageal cancer [24], colorectal cancer [25], hepatocellular carcinoma [26], glioma [27], ovarian cancer [28], bladder cancer [29, 30], breast cancer [31, 32], and oral cancer [8, 9]. Xie et al. found that ITGA5 is highly expressed in oral squamous carcinoma and is negatively correlated with the OS of patients [8, 9]. ITGA5 is also highly expressed in esophageal squamous carcinoma and head and neck squamous carcinoma, and it also played a role in promoting the proliferation, invasion, and migration of cancer cells [24, 33]. However, the relationship between ITGA5 and gastric cancer is not clear, and it is only predicted by bioinformatics data. Cao et al. [34] found that ITAG5 gene is highly expressed in gastric cancer, as revealed by the analysis of the bioinformatics database, revealing that it may be a potential diagnostic biomarker and therapeutic target for gastric cancer. However, no basic experimental confirmation is available, which is what it was demonstrated in the present experimental study. Our findings were consistent with these results, which demonstrated that ITGA5 expression was significantly higher in gastric cancer tissues than in the corresponding normal tissues adjacent to the cancer, and the increased ITGA5 expression was closely correlated with tumor size, lymph node metastasis, and TNM stage. Further confirmation in gastric cancer cell lines revealed that ITGA5 was also highly expressed in all these cells. The analysis of multiple bioinformatics databases revealed that patients with high ITGA5 expression have a poor prognosis. These results revealed that ITGA5 is an important gene in clinical application, since it could potentially be a promoter of gastric carcinogenesis, as well as an important biomarker in the diagnosis and treatment of gastric cancer.

ITGA5 wide expression and its role as a transmembrane glycoprotein were confirmed by immunohistochemical and immunofluorescence experiments in the present study, which revealed that it is mainly expressed in the cell membrane. Integrins transmit the extracellular signals to the cell, playing a key role in a variety of life activities. The ITGA5 gene is involved in the development of several tumors, since it affects tumor cell proliferation, invasion, migration, apoptosis, cell adhesion, angiogenesis, and other processes in colon cancer [7, 25], oral squamous carcinoma [8, 9], liver cancer [26], glioma [27], bladder cancer [29], ovarian cancer [35], small cell lung cancer [36], melanoma [37], and breast cancer [37]. Yu et al. [7] found that the ITGA5 gene promotes colon cancer cell proliferation and tumorigenesis in nude mice and reduces apoptosis, clearly demonstrating that ITGA5 accelerates colon cancer progression. Deng et al. [8] found that ITGA5 expression is upregulated in oral squamous cancer tissues, and OS is significantly shortened in patients with a highly expression of this gene. In addition, ITGA5 promotes oral squamous cancer cell proliferation migration and invasion through the epithelial-mesenchymal transition. Further study in oral squamous carcinoma by Lu, Fan et al. [9] confirmed by bioinformatics and experiments that ITGA5 expression is upregulated in oral squamous carcinoma. The knockdown of the ITGA5 gene inhibits its proliferation, migration, and invasion, promoting its progression by activating the PI3K/AKT signaling pathway, thus being a potential biomarker in the treatment of oral squamous carcinoma. Zheng et al. [36] identified TGA5 and ITGB1 as independent prognostic markers associated with OS in nonsmall cell lung cancer by bioinformatics analysis. Both mRNA and protein expression of ITGA5 are upregulated in glioblastoma, and that knockdown of the ITGA5 gene significantly inhibits the proliferation, invasion, and migration ability of glioblastoma cells, implying that ITGA5 may play a protumorigenic role in glioblastoma [27]. These studies revealed the protumorigenic role of ITGA5. However, the role of ITGA5 in gastric cancer is unclear, thus requiring a more in-depth study. Our results showed that the overexpression of the ITGA5 gene significantly increased the proliferation, invasion, and migration of MKN28 and AGS gastric cancer cells and accelerated the growth of the transplanted tumors, while opposite results were observed in the silenced group. These findings were consistent with the findings of ITGA5 in other tumors, which revealed that ITGA5 promotes the proliferation, invasion, and migration of a wide range of tumor cells.

Integrins mainly mediate cell-cell and cell-extracellular matrix adhesion, also acting as matrix molecule receptors that receive direct or indirect extracellular molecular signals, influencing gene expression through the transduction of specific signaling pathways, thus affecting the biological behavior of cells [38, 39]. Integrin-mediated signaling pathways are intricate and complex, and more and more studies revealed that they mainly include FAK, PI3K/AKT, ECM, and MAPK [40–43]. The most prominent pathways are the ones involving FAK, PI3K/AKT, and ECM, and our partial high-throughput sequencing analysis is consistent with these results, since they demonstrated that ITGA5 was mainly activated by FAK and phosphorylation of PI3K, AKT, and other signaling pathways, causing an altered cell biological behavior. In addition, the abnormal expression of ITGA5 was closely associated with adherent plaque kinase (FAK), as revealed by GSEA enrichment analysis. FAK is critical in cell signaling, since it is the hub of intra- and extracellular signaling in and out, mediating multiple signaling pathways. It can integrate signals from integrins, and it is enriched in adherent plaques, together with Src kinase coordinates the adhesion turnover, actin cytoskeleton kinetics, and cell morphology, regulating tumor cell migration and invasion [10, 11]. FAK mediates the FAK/PI3K/AKT/PKB, FAK/Ras/MAPK, FAK/STAT1, FAK/GSK, FAK/GTPase, and growth factor (TGF-*β*) signaling pathways, which in turn are interconnected, ultimately affecting protein and gene expression involved in proliferation, invasion, and migration of tumor cells [44–47]. Our findings are consistent with a previous article reporting that ITGA5 induces tumor cell proliferation and migration in pancreatic cancer through the activation of the FAK/Smad2 pathway [48]. Studies on the cisplatin resistance of esophageal cancer revealed that ITGA5 causes increased DNA loss repair and antiapoptosis through the activation of the FAK/PI3K/AKT signaling pathway [49]. The present study found that the high expression of ITGA5 activated the FAK/AKT signaling pathway and caused proliferation, invasion, and migration of gastric cancer cells through the phosphorylation of FAK/AKT, and conversely ITGA5 downregulation inhibited this pathway, resulting in a reduced cell proliferation, invasion and migration. Thus, ITGA5 promoted gastric cancer progression by activating the FAK/AKT pathway. This result has important implications in the treatment of gastric cancer, since targeting the FAK/AKT signaling pathway could represent a new treatment approach.

This study has some limitations. Firstly, the effect of ITGA5 gene expression on gastric cancer was only supported by bioinformatics data, lacking clinical follow-up data, while a large clinical database is needed. Secondly, our results found that the ITAG5 gene induced proliferation, invasion, and migration of gastric cancer, promoting the formation of a subcutaneous tumor in nude mice, since our study only considered a subcutaneous tumor model. Third, little is known regarding the mechanism used by ITGA5 to promote gastric cancer progression, and our results were based on previous research reports, bioinformatics prediction data, and high-throughput sequencing data, all revealing that ITGA5 might affect gastric cancer progression through the FAK/AKT signaling pathway *in vivo* and *in vitro*. However, the experiments were not confirmed by the use of pathway inhibitors or agonists. Thus, the mechanisms involved in gastric carcinogenesis and progression are complex processes that need to be further explored.

In conclusion, our results indicate that ITGA5 is highly expressed in human gastric cancer and cell lines and has protumorigenic effects. ITGA5 gene regulates the proliferation, invasion and migration of gastric cancer cells through the phosphorylation of FAK and AKT, being a potential biomarker. Therefore, our study provides a useful basis for the clinical diagnosis, treatment, and prognosis of gastric cancer.

## Figures and Tables

**Table 1:** Correlation between ITGA5 expression and patients clinicopathological characteristics in 130 GC patients. The expression level of ITGA5 gene is closely related to the clinical parameters of gastric cancer patients, and it is significantly increased in patients with large tumor, lymph node metastasis and late TNM stage (*P* < 0.05).

Parameters	Total case (*n* = 130)	ITGA5		*P* value
		High expression (*n* = 69) (%)	Low expression (*n* = 61) (%)	
Age (years)				0.8606
≤60	66	36 (54.5)	30 (45.5)	
>60	64	33 (51.6)	31 (48.4)	
Gender				>0.9999
Male	68	36 (52.9)	32 (47.1)	
Female	62	33 (53.2)	29(46.8)	
Tumor size				0.0456
≥5 cm	49	32 (65.3)	17 (34.7)	
<5 cm	81	37 (45.7)	44 (54.3)	
Lymphatic node metastasis				0.0216
Positive	72	45 (62.5)	27 (37.5)	
Negative	58	24 (41.4)	34 (58.6)	
Histological typing				0.8523
Hypodifferentiated or undifferentiated	43	22 (51.2)	21 (48.8)	
Moderately differentiated or highly differentiated	87	47 (54.0)	40 (46.0)	
TNM stage				0.0042
I + II	51	19 (37.3)	32 (62.7)	
III + IV	79	50 (63.3)	29 (36.7)	

Note: The values are statistically significant (*P* < 0.05). The 8th TNM Classification of Malignant Tumors proposed by the AJCC/UICC.

## Data Availability

The data used to support the findings of this study are included within the article.
